# A First-In-Human Dose-Escalation Phase I Study of Basroparib, a Tankyrase Inhibitor, in Patients with Advanced-Stage Solid Tumors

**DOI:** 10.1158/2767-9764.CRC-25-0502

**Published:** 2025-10-06

**Authors:** Christopher H. Lieu, Heinz-Josef Lenz, Al B. Benson, Xue Meng, Uk-Il Kim, Song Hyun Kim, Kyungjin Kim, Moo Je Sung

**Affiliations:** 1 University of Colorado Cancer Center, Aurora, Colorado.; 2 USC Norris Comprehensive Cancer Center, Los Angeles, California.; 3 Robert H. Lurie Comprehensive Cancer Center of Northwestern University, Chicago, Illinois.; 4 ST Pharm Co., Ltd., Seoul, Republic of Korea.

## Abstract

**Purpose:**

The pathogenesis of colorectal cancer is largely driven by mutations of the tumor suppressor gene *APC* (adenomatous polyposis coli) that lead to aberrant activation of the β-catenin–dependent (canonical) Wnt signaling pathway. We evaluated basroparib, a tankyrase-selective inhibitor targeting the Wnt/β-catenin signaling pathway, in a first-in-human phase I clinical study for safety, tolerability, pharmacokinetics, and efficacy in patients with advance-stage solid tumors.

**Patients and Methods:**

Patients enrolled to this open-label, multicenter study (NCT04505839) were treated with basroparib capsules orally once daily in 28-day cycles (21-day on and 7-day off) in dose escalation at seven dose levels (30–360 mg) following the classic “3 + 3” design.

**Results:**

Twenty-five patients (colorectal cancer, 23 patients; male sex, 48%, and median age, 58 years) were treated with basroparib. Dose-limiting toxicities and fatal treatment-related adverse events were not observed. The most commonly reported treatment-related adverse events were fatigue and nausea with mild/moderate severity. Basroparib pharmacokinetics increased less than proportionally with dose increase up to 300 mg. Among 17 patients evaluated for response, four (23.5%) had stable disease with a duration of up to 2.5 months and relative higher drug exposure compared with others. The dose of 360 mg was determined to be the maximum tolerated dose and recommended phase II dose.

**Conclusions:**

Basroparib was shown to be a safe and well-tolerated tankyrase-selective inhibitor with preliminary antitumor activity warranting further investigation.

**Significance:**

Tankyrase in the active β-catenin–dependent (canonical) Wnt signaling pathway has been a desirable but difficult target because of safety concerns. We developed a tankyrase-selective inhibitor, basroparib, and successfully introduced it in a first-in-human study. This study demonstrated a favorable safety profile and modest antitumor activity for basroparib and suggests that basroparib may play a role when combined with other rational targeted therapies.

## Introduction

Colorectal cancer is the second most common cause of cancer-related mortality both globally and in the United States, resulting in millions of lives lost each year despite advances in early detection and treatment (1). In view of the persistently poor prognosis and low long-term survival rate for advanced disease (2), treatment development has become molecular marker targeted based on growing understanding of colorectal cancer biology and its related signaling pathways.

The pathogenesis of colorectal cancers is largely driven by mutations of the tumor suppressor gene adenomatous polyposis coli (*APC*) that lead to aberrant activation of the β-catenin–dependent (canonical) Wnt signaling pathway. As a negative regulator of this Wnt/β-catenin pathway, APC plays a key role in intestinal tissue proliferation and homeostasis via degradation of β-catenin by the β-catenin phosphorylation complex (“destruction complex”) comprising APC, axin, GSK3β, and CK1α. Mutations of *APC* that destabilize the destruction complex lead to increasing levels of β-catenin and, ultimately, the expression of multiple tumorigenic gene products (3, 4).

Tankyrase enzymes, TNKS1 (also known as PARP-5a) and TNKS2 (also known as PARP-5b), are members of the poly(ADP-ribose) polymerase (PARP) family and are involved in various cellular functions, including Wnt signaling, mitosis, telomere maintenance, and glucose metabolism (5–8). In Wnt-dependent cancers (activated canonical Wnt signaling pathway), tankyrase destabilizes axin and eventually disrupts the formation of the β-catenin destruction complex that leads to increased transcriptional activation of β-catenin/*TCF*–regulated genes, leading to cancer cell proliferation (9). Blocking tankyrase activity is thought to restore the β-catenin destruction complex to decrease the amount of β-catenin and further diminish the activation of *TCF*/*LEF* transcription that leads to aberrant gene expressions (10–13). Thus, tankyrase is considered a favorable therapeutic target in various types of cancers (14, 15).

Basroparib is an orally available tankyrase-selective inhibitor highly active against TNKS1 and TNKS2 at 5.8 and 3.2 nmol/L, respectively. Successful tumor growth inhibitions up to around 64% by basroparib as a monotherapy were confirmed in multiple *in vivo* colorectal cancer xenograft models using *APC*-mutant cell lines and patient-derived colorectal cancer cells (16). Although several tankyrase inhibitors have already been discovered before basroparib, their on-target toxicity in the gastrointestinal (GI) tract has prevented them progressing to clinical stage (17, 18). Basroparib, on the other hand, has not been seen inducing any GI toxicity in either xenograft disease model in mouse or Good Laboratory Practice toxicology studies in rat and dog (16). This favorable trait is likely attributed to its selectivity to the Wnt signaling pathway by suppressing Wnt-activated gene expression while causing minimum interference to the expressions of intestinal stemness genes, intestinal proliferation genes, and anti-inflammatory genes (16).

Based on these promising preclinical data, we designed the first-in-human clinical trial to evaluate safety, tolerability, and pharmacokinetic (PK) characteristics of basroparib. Preliminary antitumor activity and biomarkers expression data were also collected and assessed.

## Patients and Methods

### Study design

This study was a phase I, open-label, dose-escalation, multicenter trial conducted in patients with advanced-stage solid tumors in three clinical sites located in the United States. A classic “3 + 3” method was utilized for patient enrollment starting from cohort 1 (30 mg) up to cohort 7 (360 mg). In each cohort, patients were given basroparib capsules orally once daily on a 21-day treatment period, followed by a 7-day off period in 28-day cycles until there seemed evidence of progressive disease, intolerable toxicity, or discontinuation or withdrawal for other reasons. A safety monitoring committee (SMC) composed of the principal investigators, sponsor’s representatives, and designated medical monitor reviewed the safety, tolerability, and PK of each dose level and delivered consensus decisions for dose escalations, maximum tolerated dose (MTD), and recommended phase II dose (RP2D). Study objectives and endpoints are provided in Supplementary Table S1.

### Patients

Male or female patients 18 years of age or older with a performance status of 0 to 2 on the Eastern Cooperative Oncology Group scale who had a histologically confirmed diagnosis of an advanced-stage solid tumor (metastatic or locally advanced and unresectable) of colorectal cancer, non–small cell lung cancer, gastric cancer, renal carcinoma (RCC), or hepatocellular carcinoma were identified and enrolled. As the Wnt signaling pathway also plays an important role in bone development and homeostasis (19), as well as in vascular morphogenesis (20), the potential physiologic effects of tankyrase inhibitors, which interfere with this pathway, remain unclear. To ensure participant safety, patients diagnosed with osteoporosis and/or had any history of retinal pathology at the time of screening were excluded from the study. Detailed inclusion and exclusion criteria are provided in Supplementary Table S2.

The study protocol was approved by Institutional Review Boards in advance of recruitment initiation, and written informed consent was obtained from all patients. The study was conducted in accordance with International Conference on Harmonization E6 Guidelines for Good Clinical Practice and the Declaration of Helsinki. Informed consent signed by each patient was collected prior to their enrollment. The study was registered on ClinicalTrials.gov (identifier: NCT04505839).

### Safety assessments

Adverse events (AE) and toxicity severity were evaluated according to the National Cancer Institute (NCI) Common Terminology Criteria for Adverse Events version 5.0. The dose-limiting toxicities were defined as any of the following AEs that are possibly, probably, or definitely related to the study drug occurring during cycle 1 (28 days): any death not clearly due to the underlying disease or extraneous causes; neutropenic fever; grade 4 or higher neutropenia or thrombocytopenia over 7 days; grade 3 or higher thrombocytopenia with clinically significant bleeding; for patients with hepatic metastases, aspartate aminotransferase (AST) or alanine aminotransferase (ALT) >8-fold of the upper limit of normal range (ULN) or AST or ALT >5-fold of ULN for 14 days or longer; for patients with hepatic metastases, grade 3 or higher elevation in AST and/or ALT >5-fold of ULN of any duration; and AST or ALT >3-fold of ULN and concurrent total bilirubin >2-fold of ULN of any duration without initial findings of cholestasis (elevated alkaline phosphatase, e.g., findings consistent with the Hy law or US Food and Drug Administration definition of potential study drug–induced liver injury). Medical Dictionary for Drug Regulatory Activities version 26.1 was used for safety coding.

### PK of basroparib

Blood samples for PK analysis were collected with K_2_EDTA tubes in serial before dose and at 0.25, 0.5, 1, 1.5, 2, 3, 4, 6, 8, 12, and 24 hours after dose on cycle 1 day 1 and before dose and at 1, 2, 3, 4, and 6 hours after dose on cycle 1 day 8 and before dose on days 1 and 15 of cycle 2 and beyond. Plasma concentrations of basroparib and its major metabolite, STP06-1007, were determined using the validated high-performance liquid chromatography/tandem mass spectrometry method at the bioanalytic laboratory (Celerion, Inc.). The analytic range (lower limit of quantitation – upper limit of quantitation) for basroparib and STP06-1007 was 0.05 to 250 ng/mL. Non-compartmental PK parameters were calculated using Phoenix WinNonlin version 8.4.0 (Certara).

### Antitumor activity

Antitumor activity evaluation was conducted using tumor imaging data by CT scan or MRI obtained at screening visit and within 1 week prior to the start of each odd-numbered cycle from cycle 3 and beyond and at the end-of-treatment visit. Best overall response, progression-free survival (PFS), and disease control rate were evaluated following modified Response Evaluation Criteria in Solid Tumours version 1.1.

### Pharmacodynamic biomarker analysis

The mutation status of mechanism-related (*APC*) and common oncogenes (*KRAS*, *BRAF*, and *NRAS*) was obtained from fresh tumor biopsies or latest medical records in patients with colorectal cancer. Mechanism-related biomarker protein (axin and β-catenin) expression levels were evaluated in archived or freshly prepared formalin-fixed, paraffin-embedded tissue blocks collected at pre- and posttreatment visits using the immunohistochemistry method. Well-known cancer biomarker [carcinoembryonic antigen (CEA) for colorectal cancer and non–small cell lung cancer, α-fetoprotein for HCC, carbohydrate antigen 19-9 for gastric cancer, and vascular endothelial growth factor for RCC] levels were also analyzed throughout the study.

### Statistical considerations

Study design considerations were to obtain preliminary safety, PK, and efficacy information; hence, no formal hypotheses were tested in this study. All patients who received at least one dose of basroparib were included for safety analysis. Appropriate descriptive statistics were used for summarizing analyzed data. PFS was measured in days as the date of first event – the date of first dose + 1. The median PFS was estimated using the Kaplan–Meier method. All tests of treatment effects were conducted at a two-sided α level of 0.05 and all confidence intervals (CI) were given at a two-sided 95% level.

## Results

### Demographic characteristics

A total of 25 patients were enrolled in the study from July 2020 to February 2023 at three clinical sites in the United States. Patient population characteristics at baseline are presented in Table 1 and Supplementary Table S3. The overall median age was 58 years (54 years for 13 female patients). The majority race was White (76%) and then Asian (12%). Most patients were at Eastern Cooperative Oncology Group grade 0 (28%) or grade 1 (68%) at baseline. Patients with colorectal cancer constituted the majority proportion (23 patients, 92%) of malignancies in this study. Enrolled patients had previously experienced one to 12 systemic therapies (median at 4, including radiotherapy).

**Table 1.** Patient demographic and baseline characteristics (*N* = 25).

Characteristic	Result
Age (years), median (range)	58 (41–73)
Sex, *n* (%)	​
Female	13 (52)
Male	12 (48)
Ethnicity, *n* (%)	​
Hispanic	3 (12)
Non-Hispanic	22 (88)
Race, *n* (%)	​
White	19 (76)
Black/African American	2 (8)
Asian	3 (12)
Other	1 (4)
ECOG, *n* (%)	​
0	7 (28)
1	17 (68)
2	1 (4)
Cancer type, *n* (%)	​
Colorectal cancer	23 (92)
RCC	2 (8)
No. of prior systemic therapies, median (range)	4 (1–12)

*n* (%) = 100 × *n*/*N*; ECOG, Eastern Cooperative Oncology Group; *n*, number of patients in each category; *N*, number of enrolled patients; and other, recorded as Latino in the database.

### Safety, tolerability, and AEs

Twenty-four of 25 (96%) patients had at least one treatment-emergent AE. The majority of observed treatment-emergent AEs were classified as grade 1 (50%) or grade 2 (33.7%) in severity. The most frequently reported treatment-related AEs (TRAE) were fatigue (28%), nausea (12%), and lymphocyte count decrease (8%). Four TRAEs at grade 3 or higher were pancreatitis (grade 3), lipase increased (grade 4), and amylase increased (grade 4) reported in one patient from cohort 4 receiving 180 mg once daily and hypercalcemia (grade 4) in one patient from cohort 6 receiving 300 mg once daily. No grade 5 TRAEs were reported. Detailed data are summarized and presented in Table 2.

**Table 2.** Summary of AEs related to basroparib.

	Cohort 1–3 (30–120 mg, once daily) *N* = 10		Cohort 4 (180 mg, once daily) *N* = 5		Cohort 5 (240 mg, once daily) *N* = 3		Cohort 6 (300 mg, once daily) *N* = 3		Cohort 7 (360 mg, once daily) *N* = 4		Safety set (All dosage levels) *N* = 25	
TRAE, grade	1–2	≥3	1–2	≥3	1–2	≥3	1–2	≥3	1–2	≥3	1–2	≥3
Any TRAE	5 (50%)	0	2 (40%)	1 (20%)	1 (33%)	0	1 (33%)	1 (33%)	1 (25%)	0	10 (40%)	2 (8%)
Abdominal pain upper	0	0	1 (20%)	​	0	0	0	0	0	0	1 (4%)	0
Amylase increased	0	0	0	1 (20%)	0	0	0	0	0	0	0	1 (4%)
Chills	0	0	0	0	0	0	0	0	0	0	1 (4%)	0
Diarrhea	1 (10%)	0	0	0	0	0	0	0	0	0	1 (4%)	0
Erectile dysfunction	1 (10%)	0	0	0	0	0	0	0	0	0	1 (4%)	0
Fatigue	2 (20%)	0	2 (40%)	0	1 (33%)	0	1 (33%)	0	1 (25%)	0	7 (28%)	0
Headache	1 (10%)	0	0	0	0	0	0	0	0	0	1 (4%)	0
Hypercalcemia	0	0	0	0	0	0	0	1 (33%)	0	0	0	1 (4%)
Lipase increased	0	0	0	1 (20%)	0	0	0	0	0	0	0	1 (4%)
Lymphocyte count decreased	0	0	1 (20%)	0	0	0	0	0	1 (25%)	0	2 (8%)	0
Nausea	1 (10%)	0	0	0	1 (33%)	0	0	0	1 (25%)	0	3 (12%)	0
Neuropathy peripheral	0	0	0	0	0	0	0	0	1 (25%)	0	1 (4%)	0
Pancreatitis	0	0	0	1 (20%)	0	0	0	0	0	0	0	1 (4%)
Peripheral swelling	1 (10%)	0	0	0	0	0	0	0	0	0	1 (4%)	0
Pruritus	1 (10%)	0	0	0	0	0	0	0	0	0	1 (4%)	0
Stomatitis	1 (10%)	0	0	0	0	0	0	0	0	0	1 (4%)	0

Data are presented in *n* (%); *n* (%) = 100 × *n*/*N*; *n*, number of patients who reported TRAE; *N*, number of patients included in safety set; and TRAEs included “possibly related,” “probably related,” and “related” AEs.

Except for a relatively noticeable mean body weight decrease at higher dosage levels (3.5 kg in cohort 6 at 300 mg once daily and 10.6 kg in cohort 7 at 360 mg once daily), no other apparent trends were seen in physical examinations, laboratory tests, dual-energy x-ray absorptiometry scan, and other evaluations, including ophthalmology evaluations. No dose-limiting toxicities were reported at any dose level throughout this study. The dose of 360 mg was determined by the safety monitoring committee to be the MTD and RP2D of basroparib after review all data available.

### PK analysis

Key PK parameters of basroparib are summarized in Table 3. The maximum concentrations (C_max_) of the study drug in blood were reached around 3 to 4 hours (median) after oral administration of basroparib at all dose levels on both day 1 and day 8 (steady state). The peak (C_max_) and overall exposure (AUCs) increased along with basroparib dose increase up to 300 mg in a less than proportional manner. However, exposure following 360 mg dose was slightly lower than that observed with 300 mg dose. Basroparib was eliminated from human body at a moderate rate, with mean half-life (t_1/2_) ranging from approximately 6 to 9 hours, except for the 60 mg dose (cohort 2), which had a half-life (t_1/2_) value of 11 hours and exhibited high variability (±SD of 5.33).

**Table 3.** PK parameters of basroparib.

	Day 1					Day 8		
Dosage level	C_max_ (ng/mL)	T_max_ (hours)	AUC_0–6_ (hours × ng/mL)	AUC_last_ (hours × ng/mL)	t_1/2_ (hours)	C_max,ss_ (ng/mL)	T_max,ss_ (hours)	AUC_0–6,ss_ (hours × ng/mL)
Cohort 1 (30 mg, once daily) *N* = 3	720.7 (37.01)	3.0 (3, 3)	2,464.7 (50.60)	4,845.4 (50.38)	6.0 (±3.69)^a^	789.0 (5.20)	3.0 (3, 6)	2,584.9 (22.71)
Cohort 2 (60 mg, once daily) *N* = 4	968.3 (30.69)	3.0 (3, 4)	3,169.7 (44.98)	6,415.2 (69.39)	11.0 (±5.33)^b^	1,046.3 (49.51)	3.5 (3, 6)	3,703.0 (23.05)
Cohort 3 (120 mg, once daily) *N* = 3	1,532.4 (68.26)	3.0 (3, 24)	5,425.0 (51.50)	15,835.9 (25.14)	7.6 (±0.42)^a^	1,526.2 (44.31)	3.0 (3, 4)	6,447.2 (65.26)
Cohort 4 (180 mg, once daily) *N* = 5	2,345.5 (68.30)	3.0 (2, 3)	8,233.8 (67.46)	16,504.6 (61.80)	7.7 (±3.13)	2,833.1 (105.02)	3.0 (3, 4)	10,506.4 (121.25)
Cohort 5 (240 mg, once daily) *N* = 3	2,417.1 (54.86)	3.0 (2, 6)	7,622.5 (57.67)	15,893.0 (18.35)	6.0 (±0.39)^a^	3,139.9 (86.00)	3.0 (3, 4)	10,509.6 (74.82)
Cohort 6 (300 mg, once daily) *N* = 3	2,894.1 (115.67)	3.0 (2, 4)	10,968.4 (109.20)	24,221.3 (103.59)	6.2 (±2.70)^a^	2,933.8 (79.18)	3.0 (2, 4)	16,803.6 (10.54)^a^
Cohort 7 (360 mg, once daily) *N* = 4	2,779.1 (46.78)	3.5 (3, 6)	9,022.0 (45.09)	19,692.3 (32.54)	5.8 (±0.94)	2,289.7 (70.09)	4.0 (2, 6)	7,472.7 (103.39)

AUC_0–6_, AUC_last_, C_max_, AUC_0–6,ss_, and C_max,ss_ are presented as geometric mean (geometric coefficient of variation%); T_max_ and T_max,ss_ are presented as median (min, max); and half-life (t_1/2_) is presented as arithmetic mean (±SD).

Abbreviation: *N*, number of patients.

a Two patients.

b Three patients..

Following consecutive daily dosing of basroparib, notable changes in basroparib AUC_0–6_ (1.4- to 1.7-fold) and C_max_ (1.2- to 1.6-fold) were seen on day 8 compared with day 1 at 180 to 240 mg dose levels. For the major metabolite STP06-1007, the changes in AUC_0–6_ were 1.2- to 2-fold and 1.1- to 1.5-fold in C_max_ at 30 to 240 mg dose levels.

The metabolite–parent ratios for major metabolite STP06-1007 were consistently small and variable with no clear trend between doses on both day 1 and day 8. Mean values ranged from 0.1 to 0.4 for AUCs and 0.1 to 0.3 for C_max_.

High inter-subject variations were seen in most PK parameters for basroparib and its metabolite and considered because of the low number of patients in each cohort (*n* = 2–5).

### Antitumor activity

**Figure 1.**
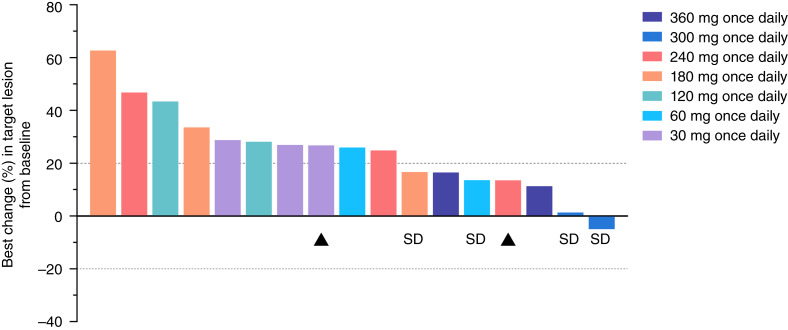
Best change (%) in target lesion from baseline. Note: “▲” marks patients with RCC; SD, stable disease in overall evaluation following Response Evaluation Criteria in Solid Tumours 1.1.

**Figure 2.**
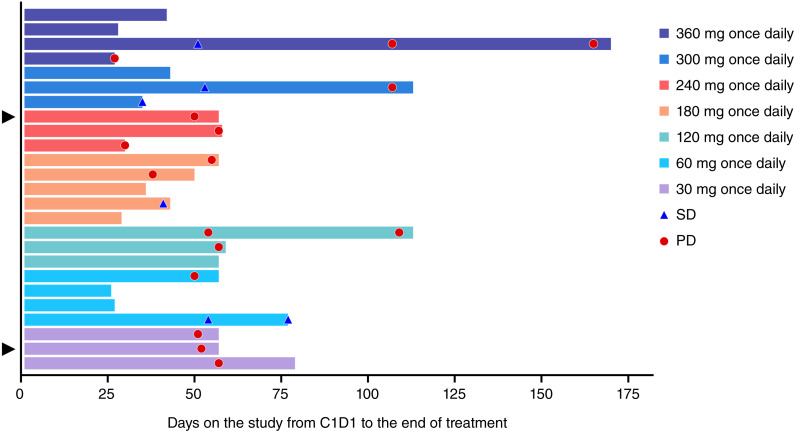
Treatment duration of all enrolled patients. Note: “▲” marks patients with RCC. “Stable Disease (SD)” and “Progressing Disease (PD)” evaluations are based on target lesions only in this graph. C1D1, cycle 1 day 1.

A total of 17 evaluable patients were included in the pharmacodynamic population. Best overall response of stable disease was seen in four patients (Fig. 1), with the change (%) from baseline of the target lesion ranging from −4.93% to 16.67%, the change (%) from nadir of the target lesion ranging from −7.69% to 16.67%, and a mean treatment duration of up to 2.5 months (Fig. 2), whereas other patients experienced progressive disease. The overall disease control rate was at 23.5% (95% CI, 6.8%–49.9%) and overall median PFS was 54 days (95% CI, 50–57 days). The PFS ranged from 35 to 107 days for stable disease patients (Supplementary Table S4).

### Pharmacodynamic biomarker analysis

A total of 60 biopsy specimens from 22 of 25 patients were collected at pre- and/or post-dose time points (including seven archival blocks/slides collected from seven patients before starting administration of study drug) and 42 specimens from 19 patients were qualified (tumor content >5%) for axin and β-catenin protein expression level evaluation using the immunohistochemistry  method.

Slight changes in mean staining intensity scores compared with baseline (screening) were observed for axin (increased in overall and cohorts 5 and 6) and β-catenin (decreased in tumor cell nuclear in overall and in cohorts 1, 3, 6, and 7), respectively (Supplementary Table S5). At individual level, paired data at pre- and post-dose time points were obtained from eight patients (cohorts 1 and 5–7: one patient from each cohort; cohorts 3 and 4: two patients from each cohort); three patients had increased axin staining intensity score by 1 level up compared with baseline, and three patients had decreased β-catenin staining intensity score by 1 to 2 levels down compared with baseline. Unfortunately, the overall changes in axin and β-catenin staining intensity did not show any notable correlations with antitumor activity or oncogene status because of the limited sample size.

Decreases in mean CEA level values from baseline were seen relatively bigger in cohort 6 (300 mg once daily, 110.20 ± 158.816 ng/mL on cycle 2 day 1) and in cohort 7 (360 mg once daily, 77.00 ± 255.731 ng/mL on cycle 1 day 15 and 4.5 ± 0 ng/mL on cycle 2 day 1) in comparison with other dose levels. Trends in mean vascular endothelial growth factor levels could not be identified because of the limited number of patients with RCC enrolled in this study (*n* = 2).

The mutation status of *APC* (16 patients, 69.6%), *KRAS* (12 patients, 52.2%), *BRAF* (three patients, 13.0%), and *NRAS* (two patients, 8.7%) was obtained or partially obtained from 23 patients with colorectal cancer. *APC*/*KRAS* dual mutations were seen in nine patients. Both patients with *BRAF* mutation also had co-existing *APC* mutation. Correlation between oncogene and antitumor activity was not determined because of limited sample size.

## Discussion

As an orally available tankyrase-selective inhibitor that modulates the *APC* mutation–induced canonical Wnt signaling pathway by suppressing tankyrase activities, basroparib was well tolerated in this first-in-human phase I clinical trial at multiple dosage levels up to 360 mg once daily, which is equivalent to two-fold of the highest non-severely toxic dosage tested in dog, the most sensitive species evaluated in preclinical toxicity studies (16). On-target toxicities in the GI tract were limited to nausea (three grade 1–2 TRAEs at 60, 240, and 360 mg) and pancreatitis (one grade 3 TRAE at 180 mg) reported in four different patients treated at four different dose levels, respectively. No TRAEs in musculoskeletal and connective tissue disorders were observed. Dual-energy x-ray absorptiometry scans also did not show any clinically meaningful changes in paired T-score and *Z*-score among all evaluable patients throughout the study even though bone loss concerns were raised for tankyrase inhibitors in published animal studies (21, 22).

Relatively limited growth increases in target lesions (−4.93% to 16.49%) were seen in evaluable patients in cohort 6 (300 mg) and cohort 7 (360 mg) up to cycle 3 day 1, which are consistent with the relative larger decreases in the CEA level in patients receiving higher doses. This observation suggested that CEA could be a reliable biomarker of response. However, the exposure–effect correlation remains unclear considering that basroparib presented a less-than-proportional dose exposure profiled in PK (even potential saturation at the 360 mg once daily level).

Although overall changes in axin and β-catenin were minimal, one patient (03-014, *APC*^+^) showed both increased axin staining intensity score (1 level up) and decreased β-catenin staining intensity score in nuclear (1–2 level down). Unfortunately, this patient’s condition deteriorated before tumor imaging could be performed, preventing confirmation of any link between biomarker changes and tumor response.

Some intriguing biomarkers of response were also discovered when taking a closer look at the patients experiencing stable disease on the study (Table 4). One patient (01-007) had a confirmed *APC* mutation with co-existing *KRAS* mutation and showed a 4.93% decrease in target lesions, suggesting that tankyrase inhibition in Wnt-dependent cancers may be more effective and warrants further investigation in future clinical trials.

**Table 4.** Summary of stable disease patients data.

		Oncogene status					Drug exposure on day 1			Drug exposure on day 8 (steady state)	
Patient ID	Dosage level	*APC*	*KRAS*	*NRAS*	*BRAF*	Best response from baseline in target lesion (%)	C_max_ (ng/mL)	AUC_0–6_ (hours × ng/mL)	AUC_last_ (hours × ng/mL)	C_max,ss_ (ng/mL)	AUC_0–6,ss_ (hours × ng/mL)
01-002	Cohort 2, 60 mg once daily	NA	NA	NA	NA	13.59%	883	3,050	4,960	744	3,200
02-002	Cohort 4, 180 mg once daily	NA	+	−	−	16.67%	4,130	16,300	40,600	6,640	27,300
01-007	Cohort 6, 300 mg once daily	+	+	−	−	−4.93%	3,890	15,900	47,800	3,820	15,600
03-012	Cohort 6, 300 mg once daily	NA	NA	NA	+	1.32%	6,050	20,800	32,000	4,970	18,100

None of these patients provided evaluable biopsy specimens for axin and β-catenin expression level assessment.

Abbreviations: NA, not available; +, mutant type; −, wild type.

In addition, three of four patients with stable disease presented relatively higher drug exposure (AUC_last_ > 32,000 hours × ng/mL and C_max_ > 3,890 ng/mL on day 1 and AUC_0–6_ > 15,000 hours × ng/mL on day 1 and day 8) compared with all other patients. Furthermore, two serious AEs, which were considered possibly related to basroparib, were both reported from patients with stable disease, patient B (pancreatitis, grade 3) and patient C (hypercalcemia, grade 4). The possibility of a correlation between these safety events and high drug exposure is worth noting. However, considering that high inter-subject variability was observed in the PK profiles across all evaluated dose levels because of a limited number of patients, drug exposure differences should be further assessed with caution. Considering the possible saturation in drug exposure at the highest dosing level, 360 mg was selected as the MTD and RP2D.

Based on these data, we suspect that less-than-optimal drug exposure is possibly one of many factors that led to current study outcomes, likely limiting its potential in antitumor activities. In addition, according to published data, tankyrase inhibitors were found to be more effective in a supportive role in combination with other targeted therapies (such as CDK4, MEK, PI3K, and EGFR inhibitors) instead of as monotherapy (23–25). Recently released nonclinical studies (26) of basroparib/MEK inhibitor combinations have elucidated new directions for further development.

In conclusion, basroparib has demonstrated a favorable safety profile and potential strategies for further development during this clinical study. We believe that a comprehensive understanding of basroparib PK characteristics may provide a foundation for further exploration in future studies as a monotherapy and also in combination with other anticancer treatments.

## Supplementary Material

Supplementary Table S1 Supplementary Table S1. Study Objectives and Endpoints

Supplementary Table S2 Supplementary Table S2. Inclusion and Exclusion Criteria of Study Participants

Supplementary Table S3 Supplementary Table S3. Representativeness of Study Participants

Supplementary Table S4 Supplementary Table S4. Summary of Progression-free Survival for Stable Disease Patients

Supplementary Table S5 Supplementary Table S5. Summary of Axin and β-Catenin Staining Intensity Scores

## Data Availability

The data generated in this study are not publicly available because of information that could compromise patient privacy and consent but are available upon reasonable request from the corresponding author.
