# Supplementary material for: A First-In-Human Dose-Escalation Phase I Study of Basroparib, a Tankyrase Inhibitor, in Patients with Advanced-Stage Solid Tumors
Source: Cancer Res Commun. 2025 Oct 6;5(10):1771–8. doi: 10.1158/2767-9764.CRC-25-0502 (PMC12498271; doi:10.1158/2767-9764.CRC-25-0502)
Supplement: Supplementary Table S1 — Study Objectives and Endpoints [file crc-25-0502_supplementary_table_s1_suppst1.docx]

**Supplementary Table S1. Study Objectives and Endpoints**

| **Study Objectives** | **Endpoints** |
| --- | --- |
| Primary Objective   - To evaluate the safety and tolerability of STP1002 by defining the MTD and determining the RP2D in patients with advanced-stage solid tumors   Secondary Objectives   - To evaluate different doses of STP1002 as monotherapy in terms of dose-limiting toxicities (DLTs); adverse event (AE) occurrence, type, and severity grades according to the National Cancer Institute Common Terminology Criteria for Adverse Events (NCI-CTCAE) version 5.0 of treatment-emergent adverse events (TEAEs); serious adverse events (SAEs); deaths; and laboratory assessments - To determine the PK of STP1002 in patients with advanced-stage solid tumors - To observe and record the antitumor activity of STP1002 by objective radiographic assessment   Exploratory Objectives:   - To observe and record biomarkers of the biological effects of STP1002 | Primary Endpoint:   - The MTD based on the number of patients experiencing ≥1 DLT within the first treatment cycle (28 days) after the administration of STP1002   Secondary Endpoints:   - Number and severity of TEAEs, treatment-related AEs, and SAEs for all dose groups according to the NCI CTCAE v5.0 - Additional safety data: incidence and type of AEs (all grades as per NCI-CTCAE v5.0), (related and unrelated) treatment-emergent SAEs, TEAEs of NCI-CTCAE with ≥ Grade 3, related TEAEs ≥ Grade 3, and TEAEs leading to permanent treatment discontinuation; laboratory tests graded by NCI-CTCAE; vital signs; 12-lead ECG changes; physical examinations including change in body weight; evaluations of Eastern Cooperative Oncology Group Performance Status (ECOG PS); and incidence and reasons for deaths including deaths within 30 days after the last dose of investigational drug - Beta-CrossLaps (Beta-CTX) levels during the treatment phase - Pharmacokinetic parameters, including AUC, C_max_, T_max_, and half-life (t_1/2_) - Antitumor activity as assessed using best overall response (BOR), progression-free survival (PFS), and disease control rate (DCR) by modified Response Evaluation Criteria in Solid Tumor (RECIST) 1.1 criteria. - Duration of response (DOR) for 6 months after a confirmed response   Exploratory Endpoints:   - Evaluation of Axin and β-catenin level in patients with colorectal cancer (CRC) - Evaluation of cancer biomarkers [e.g., serum carcinoembryonic antigen (CEA) for CRC and non-small cell lung cancer (NSCLC), alpha-fetoprotein (AFP) for hepatocellular carcinoma (HCC), CA19-9 for gastric cancer (GC), and vascular endothelial growth factor (VEGF) for renal cell carcinoma (RCC)] - Evaluation of the relationship between gene alterations and response in colorectal cancer |
