# Supplementary material for: A First-In-Human Dose-Escalation Phase I Study of Basroparib, a Tankyrase Inhibitor, in Patients with Advanced-Stage Solid Tumors
Source: Cancer Res Commun. 2025 Oct 6;5(10):1771–8. doi: 10.1158/2767-9764.CRC-25-0502 (PMC12498271; doi:10.1158/2767-9764.CRC-25-0502)
Supplement: Supplementary Table S2 — Inclusion and Exclusion Criteria of Study Participants [file crc-25-0502_supplementary_table_s2_suppst2.docx]

**Supplementary Table S2. Inclusion and Exclusion Criteria of Study Participants**

| **Inclusion Criteria** | **Exclusion Criteria** |
| --- | --- |
| To be eligible to participate in this study, an individual must meet all of the following criteria:   1. Male or female aged over 18 years at the time of signing the ICF 2. Advanced-stage solid tumor (metastatic or locally advanced and unresectable) with histologically confirmed diagnosis of CRC, NSCLC, GC, RCC, or HCC 3. Measurable lesion(s) according to RECIST 1.1 criteria 4. Performance status of ≤ 2 on the Eastern Cooperative Oncology Group (ECOG) scale 5. Ability to swallow capsules 6. Willingness and ability to comply with scheduled visits, treatment plans, laboratory tests, procedures, and lifestyle requirements 7. Received last chemotherapy, biologic, or investigational therapy at least 4 weeks prior to first dosing of study treatment 8. Has received or is intolerant to all standard of care treatment options with known clinical benefit 9. Life expectancy of more than 3 months 10. Adequate hematological, hepatic and renal function as defined below:  - Absolute neutrophil count (ANC) ≥ 1.5×10^9^/L, hemoglobin ≥ 9 g/dL, platelets ≥ 100×10^9^/L - Bilirubin ≤ 1.5× upper limit of normal (ULN), AST and ALT ≤ 5×ULN if documented liver metastases, AST and ALT ≤ 3×ULN without liver metastases - Creatinine clearance ≥ 50 mL/min (by Cockcroft-Gault formula)  1. For women of childbearing potential, a negative serum pregnancy test performed within 7 days prior to start of treatment 2. For women of childbearing potential: agreement to remain abstinent (refrain from heterosexual intercourse) or use of contraceptive methods that result in a failure rate of < 1% per year during the treatment period and for at least 90 days after the last study treatment. A woman is considered to be of childbearing potential if she is post-menarcheal, has not reached a postmenopausal state (≥ 12 continuous months of amenorrhea with no identified cause other than menopause), and has not undergone surgical sterilization (removal of ovaries and/or uterus). Examples of contraceptive methods with a failure rate of < 1% per year include bilateral tubal ligation, male sterilization, hormonal contraceptives that inhibit ovulation, hormone-releasing intrauterine devices and copper intrauterine devices. The reliability of sexual abstinence should be evaluated in relation to the duration of the clinical trial and the preferred and usual lifestyle of the patient. Periodic abstinence (e.g., calendar, ovulation, symptothermal, or postovulation methods) and withdrawal are not acceptable methods of contraception. 3. For men with reproductive potential: agreement to remain abstinent (refrain from heterosexual intercourse) or use contraceptive measures and agreement to refrain from donating sperm, as defined below: with female partners of childbearing potential or pregnant female partners, men must remain abstinent or use a condom during the treatment period and for at least 90 days after the last dose of study treatment. Men must refrain from donating sperm during this same period. The reliability of sexual abstinence should be evaluated in relation to the duration of the clinical trial and the preferred and usual lifestyle of the patient. Periodic abstinence (e.g., calendar, ovulation, symptothermal, or postovulation methods) and withdrawal are not acceptable methods of contraception. 4. Ability to understand and willingness to sign a written informed consent document | An individual who meets any of the following criteria will be excluded from participation:   1. Received treatment within the last 28 days with a drug that has not received regulatory approval for any indication at the time of study entry 2. Major surgery within the last 28 days prior to the first dose of investigational drug 3. Prior radiation therapy within 14 days prior to study Cycle 1 Day 1 and/or persistence of radiation-related adverse effects. However, palliative radiation therapy (as long as it does not involve target lesions) is permitted on the study. 4. Concurrent treatment with any anticancer agent 5. Currently taking either strong CYP inhibitors or inducers 6. Known brain metastases, uncontrolled seizure disorder, or active neurologic disease 7. Significant cardiovascular impairment which is defined as having one of the following:  - Baseline QTcF ≥ 480 msec - History of Torsades de Pointes or serious cardiac arrhythmia requiring medication - Left ventricular ejection fraction < 50% or lower institutional limit of normal as determined by echocardiogram or other appropriate method - Congestive heart failure (≥ New York Heart Association class 3 heart failure) - History of unstable angina or myocardial infarction within the past 6 months - History of stroke/transient ischemic attack within the past 6 months  1. Pregnant or nursing 2. Known HIV infection, active hepatitis C and/or hepatitis B infection 3. Known bleeding disorder or coagulopathy 4. Judged to be unsuitable to participate in the study by the Investigator 5. Known history of difficulty swallowing, malabsorption or other conditions that may reduce the absorption of investigational drug 6. Active drug or alcohol abuse or history of alcohol or drug abuse during the last two years. 7. Diagnosis of osteoporosis at the time of the screening (T-score of less than -2.5 by DEXA scan) 8. Any history of retinal pathology including diabetic retinopathy, macular degeneration, or other retinal degenerative disease |
