# Supplementary material for: A First-In-Human Dose-Escalation Phase I Study of Basroparib, a Tankyrase Inhibitor, in Patients with Advanced-Stage Solid Tumors
Source: Cancer Res Commun. 2025 Oct 6;5(10):1771–8. doi: 10.1158/2767-9764.CRC-25-0502 (PMC12498271; doi:10.1158/2767-9764.CRC-25-0502)
Supplement: Supplementary Table S3 — Representativeness of Study Participants [file crc-25-0502_supplementary_table_s3_suppst3.docx]

**Supplementary Table S3. Representativeness of Study Participants**

| **Cancer type(s)/subtype(s)/stage(s)/condition** | Advanced-Stage Solid Tumor |
| --- | --- |
| **Considerations related to:** | |
| **Sex** | In the United States in 2025, an estimated 1,053,250 new cancer cases will occur in male, slightly exceeding the number of estimated new cases in female (988,660). Among these, more than 90% are expected to be solid tumors in both sexes^1^. |
| **Age** | Around 88% of individuals diagnosed with cancer in the US are 50 years or older, and 59% are 65 or older^1^. |
| **Race/ethnicity** | In the US, American Indian and Alaska Native (AIAN) populations have the highest cancer incidence and mortality, followed by non-Hispanic White and Black populations. Asian American/Pacific Islander populations have the lowest cancer incidence and mortality. |
| **Geography** | Cancer remains to be the second leading cause of death in the US overall and the leading cause in the population younger than 85 years. |
| **Overall representativeness of this study** | The age distribution of our study is 58 (41-73) years. It is similar to those in other advanced solid tumor studies and slightly lower than the approximate median age at cancer diagnosis in US overall.  Our study presented a balanced sex ratio of 52% female and 48% male.  Study population was composed with mainly White patients (76%), followed by Asian (12%), and African American (8%). Four percent were of self-reported ‘Other’ race.  In terms of ethnicity, non-Hispanic patients (88%) comprised the majority of the population. The remaining 12% were Hispanic patients.  This study was conducted at three sites in the US, which may limit the representativeness of racial and ethnic distributions compared to broader geographic populations.  This study aimed to include patients from five different cancer types, though only colorectal cancer and renal cell carcinoma were ultimately enrolled and comprised the final study population. |

^1^ American Cancer Society. Cancer Facts & Figures 2025.  Atlanta: American Cancer Society; 2025.
