# Supplementary material for: A First-In-Human Dose-Escalation Phase I Study of Basroparib, a Tankyrase Inhibitor, in Patients with Advanced-Stage Solid Tumors
Source: Cancer Res Commun. 2025 Oct 6;5(10):1771–8. doi: 10.1158/2767-9764.CRC-25-0502 (PMC12498271; doi:10.1158/2767-9764.CRC-25-0502)
Supplement: Supplementary Table S4 — Summary of Progression-free Survival for Stable Disease Patients [file crc-25-0502_supplementary_table_s4_suppst4.docx]

**Supplementary Table S4. Summary of Progression-free Survival for Stable Disease Patients**

| **Patient ID** | **Dose Level** | **Study Visit of Event** | **Time to Event (Days)** | **Censoring Indicator** |
| --- | --- | --- | --- | --- |
| 01-002 | Cohort 2, 60 mg QD | End of Treatment | 77 | Censored (Withdrawal) |
| 02-002 | Cohort 4, 180 mg QD | End of Treatment | 43 | Censored (Adverse Event) |
| 01-007 | Cohort 6, 300 mg QD | End of Treatment | 35 | Censored (Adverse Event) |
| 03-012 | Cohort 6, 300 mg QD | End of Treatment | 107 | Progression |
