# Supplementary material for: A First-In-Human Dose-Escalation Phase I Study of Basroparib, a Tankyrase Inhibitor, in Patients with Advanced-Stage Solid Tumors
Source: Cancer Res Commun. 2025 Oct 6;5(10):1771–8. doi: 10.1158/2767-9764.CRC-25-0502 (PMC12498271; doi:10.1158/2767-9764.CRC-25-0502)
Supplement: Supplementary Table S5 — Summary of Axin and β-Catenin Staining Intensity Scores [file crc-25-0502_supplementary_table_s5_suppst5.docx]

**Supplementary Table S5. Summary of Axin and β-Catenin Staining Intensity Scores**

| **Dose Level** | **Axin** | | **β-Catenin** | | | | | |
| --- | --- | --- | --- | --- | --- | --- | --- | --- |
|  | **Baseline** | **C2D1** | **Baseline** | | | **C2D1** | | |
|  |  |  | **Membranous Intensity** | **Cytoplasmic Intensity** | **Nuclear Intensity** | **Membranous Intensity** | **Cytoplasmic Intensity** | **Nuclear Intensity** |
| Cohort 1, 30 mg QD | 2.00  (-, 1) | 1.50  (0.71, 2) | 3.00  (-, 1) | 2.00  (-, 1) | 3.00  (-, 1) | 2.00  (1.41, 2) | 2.00  (0.00, 2) | 1.50  (2.12, 2) |
| Cohort 2, 60 mg QD | 1.67  (0.58, 3) | - | 2.33  (0.58, 3) | 3.00  (0.00, 3) | 1.00  (1.73, 3) | - | - | - |
| Cohort 3, 120 mg QD | 3.00  (0.00, 4) | 2.80  (0.45, 5) | 2.50  (0.58, 4) | 2.50  (0.58, 4) | 0.50  (1.00, 4) | 1.80  (0.84, 5) | 2.80  (0.45, 5) | 0.00  (0.00, 5) |
| Cohort 4, 180 mg QD | 1.25  (0.46, 8) | 1.25  (0.50, 4) | 3.00  (0.00, 8) | 1.88  (0.83, 8) | 0.88  (1.25, 8) | 3.00  (0.00, 4) | 1.50  (0.58, 4) | 1.50  (1.73, 4) |
| Cohort 5, 240 mg QD | 1.00  (0.00, 2) | 1.50  (0.71, 2) | 3.00  (0.00, 2) | 1.00  (0.00, 2) | 1.00  (1.41, 2) | 3.00  (0.00, 2) | 1.50  (0.71, 2) | 2.00  (0.00, 2) |
| Cohort 6, 300 mg QD | 1.67  (0.58, 3) | 2.50  (0.71, 2) | 2.67  (0.58, 3) | 0.67  (1.15, 3) | 1.67  (0.58, 3) | 3.00  (0.00, 2) | 1.50  (0.71, 2) | 0.50  (0.71, 2) |
| Cohort 7, 360 mg QD | 2.50  (0.58, 4) | 2.00  (0.00, 2) | 3.00  (0.00, 4) | 0.50  (1.00, 4) | 0.75  (1.50, 4) | 1.50  (0.71, 2) | 1.50  (0.00, 2) | 0.50  (0.71, 2) |
| Overall | 1.84  (0.80, 25) | 2.00  (0.79, 17) | 2.80  (0.41, 25) | 1.68  (1.11, 25) | 1.00  (1.22, 25) | 2.35  (0.86, 17) | 1.88  (0.78, 17) | 0.88  (1.22, 17) |

Data is presented in Mean (SD, n). SD, standard deviation; n, number of observation; -, not available
